# Prevalence of microalbuminuria and its risk factors in type 2 diabetic patients

**DOI:** 10.4103/0971-4065.43690

**Published:** 2008-07

**Authors:** M. Afkhami-Ardekani, M. Modarresi, E. Amirchaghmaghi

**Affiliations:** Diabetes Research Center, Sadoughi University of Medical Sciences and Health Services of Yazd, Iran

**Keywords:** Glycosylated hemoglobin, microalbuminuria, type 2 diabetes mellitus

## Abstract

A cross-sectional study was performed from November 2005 to July 2007 to determine the prevalence of microalbuminuria and its risk factors among type 2 diabetic patients.. Two hundred and eighty-eight type 2 diabetic patients (141 males and 147 females) referred to Yazd diabetes research center were randomly recruited for the study. Microalbuminuria was detected by measuring the albumin to creatinine ratio in the early morning urine. Microalbuminuria was diagnosed if this ratio was between 30 and 300 mg/g on two occasions during three months. Prevalence of microalbuminuria was 14.2%. Chi-square analysis revealed that microalbuminuria was correlated with the diastolic blood pressure (*P* = 0.003) and the duration of diabetes (*P* = 0.000). No statistically significant correlation was found between microalbuminuria and age, sex, body mass index, levels of fasting blood sugar, glycosylated hemoglobin (HbA1c), serum triglyceride, and serum cholesterol, or systolic blood pressure. For 240 patients for whom the duration of diabetes was known from the answers in their questionnaires, logistic regression was used for analysis. Results showed that two variables including the duration of diabetes and Diastolic Blood Pressure (DBP) play a role in this model and the following Logic association was obtained: g^ (x) = -9.233 ± 0.079 DBP ± 0.114 duration according to this model, both DBP and duration of diabetes were directly correlated with microalbuminuria. Determination of the urine albumin to creatinine ratio is an easy method for screening of microalbuminuria that is suggested for all diabetic patients, especially diabetic patients with hypertension and long-term diabetes.

## Introduction

Diabetes is an important metabolic disorder worldwide and is characterized by variable degrees of insulin resistance, impaired insulin secretion, and increased glucose production.^1^ Type 2 diabetes mellitus is a leading cause of morbidity and mortality. Cardiovascular disease is the most prevalent complication and primarily accounts for the excess morbidity and mortality in diabetic patients. However, microvascular complications, such as kidney disease and retinopathy, are frequent and contribute to the total disease burden. Abnormal levels of urinary albumin occur in 30–40% of patients with type 2 diabetes and the presence of kidney disease enhances the mortality from cardiovascular disease. Microalbuminuria, an early marker of diabetic nephropathy, is an independent risk factor for cardiovascular disease. The increased levels of urinary albumin secretion may represent a more generalized vascular damage than renal microvascular injury alone.^2^ During the past decade, the incidence of end-stage renal disease has risen dramatically, primarily due to an increase in the incidence of diabetes.^3^ Glomerular hyperperfusion and renal hypertrophy occurs in the initial phase after the onset of diabetes mellitus and are reflected by an increased glomerular filtration rate. During the first five years of diabetes mellitus, the glomerular filtration rate returns to normal. After 5–10 years of type 1 diabetes mellitus, 40% of individuals begin to excrete small amounts of albumin in the urine (microalbuminuria). Microalbuminuria is defined as 30 to 300 mg/d in a 24-h collection or 30 to 300 µg/mg creatinine in a spot collection. The appearance of microalbuminuria in type 1 diabetes mellitus is a very important predictor of progression to overt proteinuria (>300 mg/d), When overt proteinuria is presented, 50% of these cases reach end-stage renal disease in 7–10 years. But in type 2 diabetes mellitus, microalbuminuria or overt nephropathy may be present at the time of diagnosis, and hypertension more commonly accompanies microalbuminuria or overt nephropathy in type 2 diabetes mellitus. Albuminuria in type 2 diabetes mellitus may be secondary to factors unrelated to diabetes mellitus such as hypertension, congestive heart failure, prostate disease, or infection.^4^,^5^ Microalbuminuria in diabetes mellitus is a risk factor for cardiovascular disease.^6^

The presence of microalbumin in the urine of persons with type 2 diabetes is perhaps the most important early signal heralding the onset of systemic vasculopathy and associated with target organ damage (the brain, the heart, and the kidneys). Microalbuminuria also identifies patients who need more rigorous cardiovascular risk management, especially more intensive blood pressure control, and strict attention to glycemic control and lipid levels.^7^

One of the central functions of the kidney is the excretion of low molecular weight, water-soluble, plasma waste products into the urine, whereas macromolecules the size of albumin and larger, are retained. The flow of the glomerular filtrate is thought to follow an extracellular route, passing through the endothelial fenestrate, then across the glomerular basement membrane, and finally through the slit diaphragm between the foot processes of podocytes. It has been recently hypothesized that microalbuminuria leading to proteinuria and end-stage renal disease is mainly due to an altered glomerular filtration barrier at the podocyte level. However, arterial hypertension and abnormalities of blood lipid concentrations and structure are also important antecedents of such complications in diabetes mellitus. Interestingly, it has been suggested that hyperglycemia, arterial hypertension, and dyslipidemia cause disorders of the albumin excretion rate by damaging the podocyte and slit diaphragm protein scaffold with overproduction of and extracellular release of oxygen radical species at the glomerular level.^8^

Bruno *et al.* followed 1253 type 2 diabetic patients over seven years and showed the progression of 3.7% of type 2 diabetic patients to overt nephropathy every year and microalbuminuria provided a risk increased by 42% as compared to normoalbuminuria.^9^

This raises the question as to which of the above statistics shows the importance of early diagnosis, treatment, and prevention of microalbuminuria in type 2 diabetic patients. As there is a high prevalence of diabetes in Yazd (14.2% of the population aged above 30 years are suffering from diabetes),^10^ we decided to evaluate the relationship between microalbuminuria and its risk factors in type 2 diabetic patients in Yazd.

## Patients and Methods

The study group comprised of 300 consecutive type 2 diabetic patients attending the Yazd diabetes research center. The cross-sectional study was performed from November 2005 to July 2007. This study was approved by the Medical Ethics Committee of Shahid Sadoughi University of Medical Sciences and Health Services of Yazd.

We completed a questionnaire including demographic data and duration of diabetes for all patients, and measured their heights and weights to estimate the body mass index. Weight was measured using the Secca scale; a body mass index <25 kg/m^2^ was considered to be normal.^11^

The blood pressure was recorded with the aid of a mercury sphygmomanometer in the right upper arm in the sitting position after five minutes rest. Patients were categorized as hypertensive patients if the systolic blood pressure was > 130 mm Hg and / or diastolic blood pressure was >85 mm Hg.^4^

A sample of blood was drawn after overnight fasting of 12 h to measure fasting blood sugar, glycosylated hemoglobin (HbA1c), serum cholesterol, and serum triglyceride levels.

Fasting blood sugar, triglyceride, and cholesterol levels were determined by enzymatic methods. HbA1c was measured by using a DS5 device; HbA1c <7% was considered to be normal.^12^ Urine samples were collected in the early morning after overnight fasting. The first morning urine specimen was assessed if evidence of urinary infection and hematuria was not seen and if the urine's specific graviity was >1.015. A diagnosis of microalbuminuria is made when the ratio of urinary albumin to creatinine is 30–300 mg/g in two out of three readings whereas macroalbuminuria is diagnosed when the same ratio is >300 mg/g. Normoalbuminuria was said to exist if this ratio was <30 mg/g (with combi-screen-9 kit with Clinitek).^4^,^5^

No further investigations were done if the result of the first urine sample indicated normoalbuminuria or macroalbuminuria, however, urine was retested over the following three months if the result indicated microalbuminuria. If the urine albumin to creatinine ratio was between 30 and 300 mg/g on two occasions over a period of three months, a diagnosis of microalbuminuria was made.

Medical history was obtained from the patients during recruitment. Treatment consisted of 75% metformin, 72% glibenclamide, 6.8% acarbose, 30% insulin alone, 7% insulin with oral hypoglycemic agents (OHAs), 34% statins, and 22% angiotensine converting enzyme inhibitors (ACEIs).

In this study, 12/300 patients were excluded because they had macroalbuminuria. Data analysis was done using the Statistical Package for Social Sciences (SPSS) for Windows version 10. Chi-square test and Logistic Regression were used to determine the correlation between microalbuminuria and its risk factors.

## Results

Two hundred and eighty-eight type 2 diabetic patients were studied (141 males and 147 females); Table 1 represents the characteristics of patients in this study. The mean age of the patients was 53.2 ± 9.9 years and the mean body mass index was 27.7 ± 4.11 kg/m^2^. The mean duration of diabetes (known for 240 patients) was 9.3 ± 6.3 years. The mean fasting blood sugar and HbA1c levels were 167.5 ± 46.5 mg/dL and 9.1 ± 2.15%, respectively. The mean serum triglyceride and cholesterol levels were 211.8 ± 88.5 and 201.7 ± 39.7 mg/dL, respectively. The mean systolic blood pressure and diastolic blood pressure were 127.4 ± 17.6 and 78.0 ± 8.6 mm Hg, respectively [Table 1]. The overall prevalence of microalbuminuria was 14.2%.

**Table 1:** Patients' characteristics

	*n*	Maximum	Minimum	Mean ± SD^1^
Age (years)	288	25	78	53.2±9.9
Duration of diabetes (years)	240	1	36	9.3±6.3
Body mass index (kg/m^2^)	288	17.43	47.27	27.7±4.11
Fasting blood sugar (mmol/L)	288	4.2	18.7	9.3±2.6
HbA1c (%)	288	4.2	16.5	9.1±2.15
Triglyceride (mmol/L)	288	0.7	8.2	2.4±1.0
Cholesterol (mmol/L)	288	2.3	8.7	5.2±1.1
Systolic blood pressure (mm Hg)	288	90	200	127.4±17.6
Diastolic blood pressure (mm Hg)	288	60	120	78.0±8.6

1 Standard deviation

First, we used the Chi-square test to analyze our results; the prevalence of microalbuminuria was not statistically different between the various age groups (*P* = 0.6) [Table 2]. The prevalence of microalbuminuria among males was 14.9% whereas it was 13.6% among females. No statistically significant correlation was found between the prevalence of microalbuminuria and the sex (*P* = 0.754) [Table 2]. The prevalence of microalbuminuria in patients with a duration of diabetes for ≤10 and >10 years was 7.3 and 28.1% respectively. A significant correlation was found between the prevalence of microalbuminuria and the duration of diabetes (*P* = 0.001) [Table 2].

**Table 2:** The relationship between microalbuminuria and different variables in diabetic patients

Variables	Albuminuria	Microalbuminuria		Normo-albuminuria		Total		*P* value
					
		*%*	*n*	*%*	*n*	*%*	*n*
Age	≤45	8	11.6	61	88.4	69	24	0.6
	45–60	21	14.1	128	85.9	149	51.7
	>60	12	17.1	58	82.9	70	24.3
	Total	41	14.2	24.7	85.8	288	100
Sex	Female	20	13.6	127	86.4	147	51	0.754
	Male	21	14.9	120	85.1	141	49
	Total	41	14.2	247	85.8	288	100
Duration of diabetes (years)	≤10	11	7.3	140	92.7	151	62.9	0.001
	>10	25	28.1	64	71.9	89	37.1
	Total	36	15	204	85	240	100
Diastolic blood pressure (mm Hg)	<85	27	11.7	210	88.6	237	82.3	0.003
	≥85	14	27.5	37	72.5	51	17.7
	Total	41	14.2	247	85.8	288	100

The prevalence of microalbuminuria among patients with systolic blood pressure ≥ and < 130 mm Hg was 17.5 and 10.2% respectively. There was no significant correlation between raised systolic blood pressure and microalbuminuria (*P* = 0.076). The prevalence of microalbuminuria among patients with diastolic blood pressure < and ≥85 mm Hg was 11.7 and 27.5% respectively. There was a significant correlation between microalbuminuria and raised diastolic blood pressure (*P* = 0.003) [Table 2].

The prevalence of microalbuminuria among patients with fasting blood sugar < and > 140 mg/dL was 13.9 and 14.4% respectively. There was no significant correlation between fasting blood sugar and microalbuminuria (*P* = 0.926). The prevalence of microalbuminuria among patients with HbA1c ≤ and >7% was 16.7 and 13.7% respectively. There was no significant correlation between HbA1c and microalbuminuria (*P* = 0.571).

The prevalence of microalbuminuria among patients with body mass index < and >25 kg/m^2^ was 17.9 and 12.9% respectively. No statistically significant correlation was found between microalbuminuria and body mass index (*P* = 0.272).

The prevalence of microalbuminuria among patients with triglyceride < and >200 mg/dL was 14.1 and 14.4% respectively. No statistically significant correlation was found between microalbuminuria and serum triglyceride (*P* = 0.944).

The prevalence of microalbuminuria among patients with cholesterol ≤ and >200 mg/dL was 10.5 and 18.5% respectively. No statistically significant correlation was found between microalbuminuria and serum cholesterol (*P* = 0.051), although this may not have been the case if we had studied more patients

After primary analysis of 240 patients whose duration of diabetes had been noted in their questionnaires, logistic regression was used for analysis and SPSS for Win (version 10) was used for data analysis using a logistic regression model. Results showed that two variables: the duration of diabetes and the diastolic blood pressure (DBP) play a role in this model and the following logic association was obtained: gˆ (x) = -9.233 + 0.079 DBP + 0.114 Duration

According to this model, both the diastolic blood pressure and the duration of diabetes directly correlate with microalbuminuria, although the correlation of duration with microalbuminuria is stronger than with diastolic blood pressure. Duration of diabetes (*P*=0.001) and diastolic blood pressure (*P*=0.003) increased the risk of microalbuminuria.

## Discussion

In the present study, 288 type 2 diabetic patients were studied and the overall prevalence of microalbuminuria was found to be 14.2%. A statistically significant correlation was found between the prevalence of microalbuminuria and diastolic blood pressure and the duration of diabetes.

Various epidemiological and cross-sectional studies have reported many variations in the prevalence of microalbuminuria.^13^–^17^ Vijay *et al.* reported a prevalence of 15.7% in 600 type 2 diabetic patients in Chennai.^13^ Huraib *et al.* reported a prevalence of 16.8% among 125 type 2 diabetic patients in Saudi Arabia^14^ whereas a previous study in Yazd reported a prevalence of 26.3% among 650 diabetic patients.^15^ Varghese *et al.* reported a prevalence of 36.3% in 1425 type 2 diabetic patients in in Chennai (India).^16^ Ko *et al.*^17^ reported a prevalence of 22.7% of albuminuria in 150 young diabetic patients in Hong Kong.^17^

This variation in the prevalence of microalbuminuria can be attributed to several factors such as differences in populations, the definition of microalbuminuria, the methods of measurement of microalbuminuria and urine collection etc.

Although both types 1 and 2 diabetic patients were studied in the previous study conducted in Yazd,^15^ only type 2 diabetic patients were studied in the present study. In addition, whereas one positive microalbuminuria test was decided as the criterion for microalbuminuria in the previous study,^15^ two positive microalbuminuria tests over three months were used as criteria for microalbuminuria in the present study.

Huraib *et al.* used an immunoturbidometric method for the assessment of microalbuminuria;^14^ the urine albumin to creatinine ratio was determined by using the Clinitek 100 device in the present study. Although the prevalence of microalbuminuria in the present study is lower than that reported in the previous study conducted in Yazd,^15^ it is similar to the prevalence of microalbuminuria reported by Hurib *et al.*^14^ and Vijay *et al.*^13^ This shows that two positive microalbuminuria tests over a period of three months is more valuable than just one positive microalbuminuria test.

No statistically correlation was found between the prevalence of microalbuminuria and the age of patients in the present study which was similar to findings reported by Allawi *et al.*^18^ A previous study conducted in Yazd also did not demonstrate any statistically significant correlation between microalbuminuria and the age among 650 diabetic patients.^15^ Varghese *et al.* reported a statistically significant correlation between the prevalence of microalbuminuria and the age among 1425 type 2 diabetic patients.^16^ These variations are probably related to the different distributions of patients' ages in the different studies.

In the present study, prevalence of microalbuminuria among males and females was 14.9 and 13.6% respectively. Thus, the prevalence of microalbuminuria was not statistically different for the two sexes, which was similar to the findings reported by Mather *et al.* in European diabetic patients.^19^ However, Varghese *et al.* reported an increased prevalence of microalbuminuria in Indian men compared with Indian women.^16^ This different prevalence of microalbuminuria between males and females can be due to the lower creatinine excretion in women than in men^20^ and the fact that we used the albumin to creatinine ratio to diagnose microalbuminuria.

In present study, no statistically significant correlation was found between body mass index and the prevalence of microalbuminuria which was similar to the findings reported by Allawi *et al.*^18^ However, Gall *et al.* reported that patients with higher body mass index had higher albumin excretion.^21^ Our findings may be explained by the fact that poorly controlled diabetes induces weight loss and these patients with low body mass index are at higher risk for diabetic complications and microalbuminuria.

In the present study, a good statistically significant correlation was found between the prevalence of microalbuminuria and the duration of diabetes that was consistent with findings of past studies. Huraib *et al.* in Saudi Arabia,^14^ Varghese *et al.*,^16^ and Mather *et al.*^19^ reported a significant correlation between microalbuminuria and the duration of diabetes.

In the present study, no statistically significant correlation was found between the prevalence of microalbuminuria and the fasting blood sugar or HbA1c, which was similar to findings reported by Huraib *et al.*^14^ However, Varghese *et al.* reported a correlation of the prevalence of microalbuminuria with the fasting blood sugar and with HbA1c levels.^16^ The previous study conducted in Yazd also showed that the HbA1c level was associated with microalbuminuria.^15^

In the present study, a good statistically significant correlation was found between the prevalence of microalbuminuria and the diastolic blood pressure which was similar to findings reported by Varghese *et al.*^16^ Huraib *et al.* reported a good correlation between the prevalence of microalbuminuria and hypertension.^14^ Svensson *et al.* showed that high blood pressure increased the risk of developing signs of nephropathy (*P* = 0.003).^22^ Thus, hypertension can cause microalbuminuria and hypertensive nephropathy that can accelerate the progression of diabetic nephropathy.

No statistically significant correlation was found in the present study between the prevalence of microalbuminuria and serum triglyceride and cholesterol levels, which was similar to findings reported by Varghese *et al.*^16^ Mather *et al.* also reported a statistically significant correlation between the prevalence of microalbuminuria and serum triglyceride levels.^19^ Smulders *et al.* reported that diabetic dyslipidemia (high serum triglyceride and low HDL cholesterol levels) is a predictor of rapid progression of microalbuminuria in patients with well-controlled blood pressure.^23^

Considering the high prevalence of diabetes in Iran (especially in Yazd), we suggest screening for microalbuminuria and vigorous control of blood pressure in diabetic patients to reduce future diabetic kidney disease.

In conclusion, the prevalence of microalbuminuria in this study was 14.2%, a finding that was similar to the ones reported in Saudi Arabia and India and the duration of diabetes and hypertension were associated with microalbuminuria, also consistent with past studies.
